# National survey on school-based fluoride mouth-rinsing programme in Japan: regional spread conditions from preschool to junior high school in 2010

**DOI:** 10.1111/idj.12068

**Published:** 2013-10-30

**Authors:** Karin Komiyama, Kazunari Kimoto, Katsuhiko Taura, Osamu Sakai

**Affiliations:** 1 Department of Oral Health, Kanagawa Dental UniversityYokosuka, Japan; 2 Non-profit Japanese Conference on the Promotion of the Use of Fluoride in Caries PreventionMizuho, Japan

**Keywords:** Caries prevention, school-based fluoride mouth-rinsing programme, national survey, regional disparities

## Abstract

**Aims:**

We surveyed the state of implementation of the school-based fluoride mouth-rinsing programme (S-FMR) in schools in Japan from March 2010.

**Methods:**

Questionnaires on the implementation status of S-FMR in each type of school (including preschool and kindergarten) were sent by post to the oral health administration departments of all 47 prefectures and 89 cities (18 ordinance-designated cities, 23 special wards, 41 core cities and seven public health centres in ordinance-designated cities) with public health centres.

**Results:**

The S-FMR implementation rate was low, at only 11% of all schools in Japan and only 6% of all participating school children aged 4–14 years. In many regions, the S-FMR was implemented more widely and received higher participation from children in either elementary schools and junior high schools or preschools and kindergartens.

**Conclusions:**

Inter-prefectural disparities were seen in S-FMR implementation, as some prefectures and cities did not include topical fluoride application in their health promotion plans, and some local public bodies did not include targets for fluoride mouth-rinsing. To reduce this disparity in Japan where systemic fluoride application is not performed, each local public body must consider implementing the S-FMR as a public health measure. We propose using the results of this survey as basic data for formulating S-FMR goals (numerical targets) and adopting S-FMR as a concrete measure in the second Healthy Japan 21, to be launched in the fiscal year for 2013, and within the basic matters of the Act Concerning the Promotion of Dental and Oral Health.

## Introduction

In Japan, where methods of systemic fluoride application such as water fluoridation (WF) are not performed, a school-based fluoride mouth-rinsing programme (S-FMR) was launched in Niigata Prefecture in 1970 and had expanded to all 47 prefectures in the country by 2005^1^. As for other fluoride uses in 2011, 64% of 1- to 14-year-olds received professional topical fluoride application; the market share of fluoridated dentifrice is 90% and FMR agents are not for sale as over-the-counter drugs in Japan.

The advantages of fluoride mouth-rinsing are that it can be performed simultaneously in groups, it has reliable preventive effects, it is guaranteed to be safe, it is easy to apply, it is very cost-effective and it allows for continuous group application^1^,^2^. From the position of public health characteristics and the mechanisms underlying fluoride effects, WF should be prioritised as an administration measure. As this has yet to come into practice in Japan, S-FMR is a promising next-best policy to correct health inequalities caused by dental caries^3^–^6^.

In Japan, the non-profit Japanese Conference on the Promotion of the Use of Fluoride in Caries Prevention that works to endorse fluoride mouth-rinsing and other types of topical fluoride application and to raise awareness and knowledge on systemic fluoride application, such as WF, worked together with the 8020 Promotion Foundation and the World Health Organisation (WHO) Collaborating Center for Translation of Oral Health Science to investigate adoption of the S-FMR in Japanese schools for each type of target school and prefecture as of March 2010.

## Subjects and Methods

### Subjects and survey methods

A survey on S-FMR implementation status as of March 2010 for each type of school (preschool, kindergarten, elementary school, junior high school and school for special needs education) that included two types of questionnaire was sent by post to the oral health administration departments of 47 prefectures and 89 cities (18 ordinance-designated cities, 23 special wards, 41 core cities and seven public health centres in ordinance-designated cities) with public health centres. Questionnaires were returned by e-mail, fax or post. The survey complied with the Personal Information Protection Law and the Ethics Guidelines for Epidemiological Research from the Ministry of Education, Culture, Sports, Science and Technology (MEXT) and the Ministry of Health, Labour and Welfare (MHLW), and was in full accordance with the World Medical Association Declaration of Helsinki. The research plan was explained to subjects and consent was requested for participation. Consent was obtained with a written answer from all oral health administration of 47 prefectures and 89 cities at a public health centre and informed consent from parents/guardians of the children/minors involved in S-FMR.

### Survey items

The two questionnaires included the following survey questions and the results for these items were tabulated separately for each type of school by prefecture.

Questionnaire 1 (number of schools and children participating in the S-FMR in the regions and presence or absence of infrastructure):

- Name of prefecture or special ward/ordinance-designated city and number of municipalities in each prefectural region
- Total number of schools implementing the S-FMR and number for each type of school
- Total number of children participating in the S-FMR and number for each type of school
- Total number of municipalities implementing the S-FMR and number for each type of school
- Implementation status of S-FMR in all schools for each municipality
- Inclusion of topical fluoride application in the health-promotion plans of local public bodies in accordance with the Health Promotion Act and, if included, the method for topical fluoride application. Questionnaire 2 (S-FMR methods in regions and funding party):
- Number of children participating in fluoride mouth-rinsing at each school for each type of rinse method (a–d below)
  - Frequency of fluoride mouth-rinsing at each type of school (five times a week, once a week, other)
  - Mouth-rinse fluoride concentration at each type of school (250 ppm F, 450 ppm F, 900 ppm F, other)
  - Fluoride mouth-rinsing agent used at each type of school [sodium fluoride reagent, fluoride mouth-rinse ethical drug contain 11% sodium fluoride, MIRANOL Granules, Bee Brand Medico-Dental Co., Ltd., Higashiyodogawa-ku, Osaka City, Japan and ORA-BLISS Gargle Gran, Showa Yakuhin Kako Co., Ltd., Chuo City (Special Ward), Tokyo Metropolis, Japan; In Japan ethical drugs is available only with written instructions from a doctor or dentist to a pharmacist]
  - Party or organisation responsible for financial support of the S-FMR at each type of school (public agency of the government and board of education, dental association, individual dentists, a combination of parents, relevant parties and organizations of the schools, other).

### Implementation result analysis by prefecture

We first calculated the number of participants and number of S-FMR implementing schools in each category (preschools, kindergartens, elementary schools, junior high schools and schools for special needs education) for each prefecture. We then calculated the ratio of S-FMR implementing schools among the total number of schools for each category in each prefecture, and the ratio of S-FMR participants in the age class investigated (age 4–14 years). The number of preschools was that listed in the 2009 MHLW Survey on Social Welfare Facilities, etc.^7^. The numbers of kindergartens, elementary schools, junior high schools and schools for special needs education were those listed in the fiscal year (FY) for 2009 MEXT School Basic Survey^8^. The number of junior high schools was the actual number, including the number of secondary education schools (former term 7–9th grade). For the number of preschool children, we used the number listed in the 2009 MHLW Survey on Social Welfare Facilities, etc.^9^, adding up the number of children attending preschool who were aged 4–6 years. For the number of children at kindergartens, elementary schools, junior high schools and schools for special needs education, we used the number of children attending each school by prefecture listed in the FY 2009 MEXT School Basic Survey^10^. The number of children attending kindergarten was the actual number based on a tabulation of 4- and 5-year-olds attending kindergarten according to the School Basic Survey and the number of junior high school students was the actual number, including those attending secondary education schools (former term).

For the number of schools implementing S-FMR and the number of programme participants in each prefecture, a relative frequency was calculated based on those numbers as frequencies, and the prefectures were arranged in ascending order. We then calculated the cumulative relative frequency and the inter-prefecture disparity in implementation of S-FMR^4^.

Excel 2010 was used for calculation. Implementation rates of schools and participation rates of children in S-FMR for prefectures by type of school were subjected to tests of difference between proportions.

## Results

**Figure 1:**
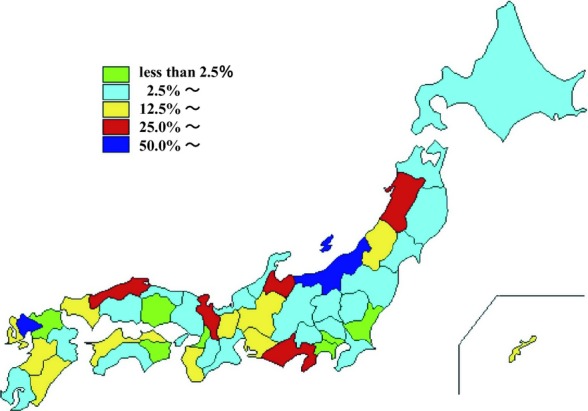
Map of the schools implementation rate of school-based fluoride mouth-rinsing programme (S-FMR) in all 47 prefectures in Japan (2010).

**Figure 2:**
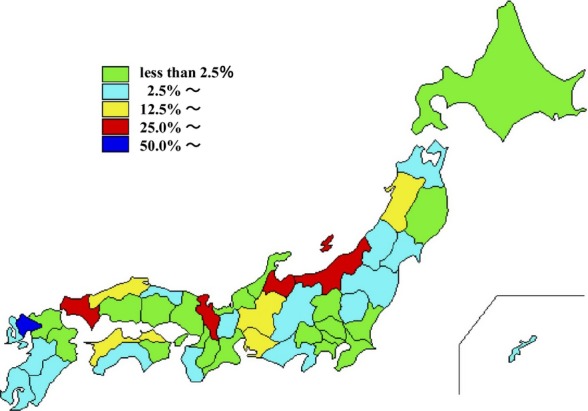
Map of the children participation rate of school-based fluoride mouth-rinsing programme (S-FMR) in all 47 prefectures in Japan (2010).

Questionnaire responses were received from all 47 prefectures and 89 cities with public health centres (response rate 100%). As of March 2010, a total of 7,479 schools and 777,596 children were participating in S-FMR in Japan. Separated by type of school, there were a total of 123,604 participants at 3,654 preschools, 65,182 participants at 1,024 kindergartens, 517,247 participants at 2,369 elementary schools, 68,095 participants at 386 junior high schools and 3,468 participants at 46 schools for special needs education (*Tables* **1** and **2**, *Figures* 1 and 2). The implementation rates found using the number of schools implementing the S-FMR by prefecture for each type of school and the total number of schools in each prefecture are shown in *Table* **1**. Participation rates found using the number of S-FMR participants and the total number of target children in each prefecture are shown in *Table* **2**. S-FMR was implemented at 11% of all schools in Japan. Looking at each type of school separately, S-FMR was being implemented at 16% of preschools, 8% of kindergartens, 11% of elementary schools, 4% of junior high schools and 5% of schools for special needs education, with the highest implementation rate being at preschools. The total percentage of children participating in S-FMR throughout Japan was very low, at only 6% of all target children. Looking at each type of school separately, 11% of children in preschools, 5% in kindergartens, 7% in elementary schools, 2% in junior high schools and 6% in schools for special needs education participated in the programme, with the highest ratio being in preschool children. In 2010, preschools made up roughly half of all the schools implementing the S-FMR programme and elementary school students accounted for about two-thirds of all participants.

**Table 1:** Number of schools implementing school-based fluoride mouth-rinsing programme (S-FMR) and implementation rate by type of school for each prefecture in Japan (2010)

Prefecture	Number of schools						Implementation rate (%)					
	
	Preschool	Kindergarten	Elementary school	Junior high school	School for special needs education	Subtotal	Preschool	Kindergarten	Elementary school	Junior high school	School for special needs education	Subtotal
Hokkaido	105	40	32	4	6	187	13.1	7.1	2.5	0.6	9.7	5.5
Aomori	21	4	9	5	0	39	4.6	3.0	2.5	2.9	0.0	3.4
Iwate	71	18	19	2	4	114	21.1	12.1	4.6	1.0	25.0	10.3
Miyagi	81	48	2	0	1	132	25.0	15.4	0.4	0.0	4.5	9.8
Akita	112	26	93	43	1	275	46.3	26.8	36.0	32.1	6.7	36.9
Yamagata	40	11	54	8	1	114	17.5	9.6	16.0	6.3	7.7	13.9
Fukushima	14	16	41	10	0	81	4.5	4.5	7.7	4.1	0.0	5.5
Ibaragi	3	1	1	0	0	5	0.7	0.3	0.2	0.0	0.0	0.3
Tochigi	3	4	82	15	0	104	0.9	2.0	20.3	8.4	0.0	9.2
Gunma	49	21	8	0	2	80	11.9	9.8	2.3	0.0	7.4	6.8
Saitama	40	19	24	4	4	91	4.6	3.0	2.9	0.9	9.8	3.2
Chiba	32	41	26	4	0	103	4.5	6.9	3.0	1.0	0.0	4.0
Tokyo	1	0	2	1	0	4	0.1	0.0	0.1	0.1	0.0	0.1
Kanagawa	17	3	0	0	0	20	1.8	0.4	0.0	0.0	0.0	0.6
Niigata	462	44	326	71	3	906	67.2	27.5	59.1	28.3	10.7	54.0
Toyama	103	23	86	18	0	230	33.9	23.2	42.2	21.4	0.0	32.7
Ishikawa	35	0	0	0	0	35	9.8	0.0	0.0	0.0	0.0	4.5
Fukui	14	2	4	0	0	20	5.5	1.6	1.9	0.0	0.0	2.9
Nagano	64	8	58	17	0	147	11.0	6.7	14.7	8.4	0.0	11.1
Yamanashi	8	3	4	2	0	17	3.4	4.0	1.9	1.9	0.0	2.7
Gifu	53	18	87	17	0	175	12.3	9.6	22.8	8.5	0.0	14.4
Shizuoka	295	157	60	8	8	528	59.4	30.2	11.2	2.7	25.8	28.1
Aichi	307	74	278	7	0	666	26.6	14.1	28.1	1.6	0.0	21.2
Mie	36	12	0	0	0	48	8.5	4.6	0.0	0.0	0.0	3.7
Shiga	43	28	27	2	0	100	17.8	14.6	11.5	1.9	0.0	12.7
Kyoto	40	10	276	5	5	336	9.2	4.3	62.0	2.5	21.7	25.1
Osaka	0	4	1	0	0	5	0.0	0.5	0.1	0.0	0.0	0.1
Hyogo	212	49	0	0	0	261	24.9	6.6	0.0	0.0	0.0	9.1
Nara	7	15	6	2	0	30	4.0	7.4	2.7	1.7	0.0	4.1
Wakayama	14	1	85	10	0	110	6.5	0.9	29.2	7.0	0.0	14.2
Tottori	43	4	2	1	0	50	23.0	10.0	1.3	1.5	0.0	10.9
Shimane	52	11	134	29	0	226	19.8	9.1	53.0	26.9	0.0	29.9
Okayama	0	5	10	0	0	15	0.0	1.5	2.3	0.0	0.0	1.1
Hiroshima	39	3	6	1	1	50	6.7	0.9	1.0	0.4	5.9	2.8
Yamaguchi	46	22	122	27	1	218	15.2	10.8	34.9	14.9	6.7	20.7
Tokushima	1	1	3	1	0	6	0.5	0.4	1.1	1.0	0.0	0.7
Kagawa	16	23	59	13	1	112	7.9	13.0	29.8	15.1	11.1	16.7
Ehime	25	9	108	14	0	156	7.8	4.8	30.7	9.4	0.0	15.3
Kochi	34	7	15	3	1	60	12.9	10.9	5.4	2.2	7.1	7.9
Fukuoka	12	11	2	0	0	25	1.4	2.2	0.3	0.0	0.0	1.0
Saga	205	68	169	24	0	466	98.6	63.0	89.4	23.3	0.0	75.5§
Nagasaki	120	42	16	6	2	186	28.9	22.2	4.0	2.8	12.5	15.1
Kumamoto	275	35	3	2	3	318	48.2	23.5	0.7	1.0	16.7	23.4
Oita	37	6	1	1	0	45	13.6	2.6	0.3	0.7	0.0	4.5
Miyazaki	171	36	10	3	1	221	45.4	26.3	3.7	2.0	6.7	23.4
Kagoshima	155	29	7	0	1	192	34.9	11.2	1.2	0.0	6.3	12.1
Okinawa	141	12	11	6	0	170	39.9	4.3	3.9	3.7	0.0	15.6
Total	3,654	1,024	2,369	386	46	7,479	16.4	7.6	10.6	3.5	4.5	10.7
Nationwide total	22,250	13,516	22,258	10,906	1,030	69,960						

§ *P* < 0.01.

**Table 2:** Number of children participating in school-based fluoride mouth-rinsing programme (S-FMR) and participation rate by type of school for each prefecture in Japan (2010)

Prefecture	Number of children						Participation rate (%)					
	
	Preschool	Kindergarten	Elementary school	Junior high school	School for special needs education	Subtotal	Preschool	Kindergarten	Elementary school	Junior high school	School for special needs education	Subtotal
Hokkaido	2,842	3,102	3,903	76	375	10,298	8.6	6.1	1.4	0.1	17.9	2.0
Aomori	608	366	2,776	1,385	0	5,135	3.7	5.1	3.6	3.3	0.0	3.6
Iwate	1,338	586	1,114	171	33	3,242	10.0	5.9	1.5	0.4	4.3	2.4
Miyagi	3,632	5,478	194	0	32	9,336	26.5	20.5	0.1	0.0	3.0	3.9
Akita	1,985	735	13,478	6,197	17	22,412	18.1	11.5	24.9	20.2	2.8	21.8
Yamagata	1,212	762	7,991	913	23	10,901	11.3	8.6	12.4	2.6	5.1	9.2
Fukushima	448	937	7,833	1,510	0	10,728	3.5	3.8	6.6	2.4	0.0	4.8
Ibaragi	135	40	383	0	0	558	0.6	0.1	0.2	0.0	0.0	0.2
Tochigi	120	424	11,230	3,876	0	15,650	0.8	1.9	9.9	6.7	0.0	7.4
Gunma	1,838	1,028	878	0	22	3,766	7.9	6.3	0.7	0.0	2.1	1.7
Saitama	1,718	2,698	7,393	980	162	12,951	4.1	3.1	1.9	0.5	5.4	1.8
Chiba	1,185	3,214	3,857	355	0	8,611	3.1	4.4	1.1	0.2	0.0	1.4
Tokyo	52	0	268	47	0	367	0.1	0.0	0.0	0.0	0.0	0.0
Kanagawa	679	422	0	0	0	1,101	1.5	0.4	0.0	0.0	0.0	0.1
Niigata	16,597	2,361	58,487	13,023	34	90,502	50.7	20.7	45.3	18.8	3.5	37.2
Toyama	3,851	921	23,738	4,067	0	32,577	24.2	16.9	39.3	13.2	0.0	28.7
Ishikawa	1,105	0	0	0	0	1,105	6.0	0.0	0.0	0.0	0.0	0.9
Fukui	453	22	233	0	0	708	3.7	0.5	0.5	0.0	0.0	0.8
Nagano	2,157	379	15,538	6,536	0	24,610	6.1	4.2	12.3	10.1	0.0	10.4
Yamanashi	204	172	274	80	0	730	1.7	3.5	0.5	0.3	0.0	0.8
Gifu	1,479	563	21,483	5,404	0	28,929	5.7	3.3	17.4	8.6	0.0	12.5
Shizuoka	11,831	9,825	14,735	3,017	1286	40,694	44.0	22.5	6.9	2.8	52.2	10.3
Aichi	10,166	5,145	91,932	2,147	0	109,390	12.3	7.6	20.9	1.0	0.0	13.5
Mie	1,037	690	0	0	0	1,727	5.1	4.3	0.0	0.0	0.0	0.9
Shiga	1,571	1,776	7,951	529	0	11,827	11.2	11.5	9.1	1.2	0.0	7.4
Kyoto	826	384	82,464	247	377	84,298	3.4	1.6	58.6	0.3	31.4	32.5
Osaka	0	415	83	0	0	498	0.0	0.4	0.0	0.0	0.0	0.1
Hyogo	8,225	2,928	0	0	0	11,153	19.7	4.9	0.0	0.0	0.0	1.9
Nara	268	1,264	1,243	34	0	2,809	2.6	8.7	1.6	0.1	0.0	1.9
Wakayama	307	59	10,937	578	0	11,881	2.7	0.9	19.2	1.9	0.0	11.2
Tottori	1,541	83	151	75	0	1,850	18.9	2.5	0.5	0.4	0.0	3.0
Shimane	1,060	254	12,241	3,407	0	16,962	11.4	6.4	31.4	16.4	0.0	23.1
Okayama	0	223	3,013	0	0	3,236	0.0	1.3	2.7	0.0	0.0	1.6
Hiroshima	1,129	39	329	9	250	1,756	3.8	0.2	0.2	0.0	25.7	0.6
Yamaguchi	1,460	1,255	29,904	3,928	78	36,625	11.0	10.1	38.2	9.7	11.0	25.2
Tokushima	6	1	66	25	0	98	0.1	0.0	0.2	0.1	0.0	0.1
Kagawa	722	848	11,589	3,231	76	16,466	8.2	7.7	20.2	11.5	13.2	15.5
Ehime	529	492	17,481	2,463	0	20,965	4.2	3.7	22.1	5.9	0.0	14.2
Kochi	704	119	895	231	82	2,031	6.8	3.7	2.2	1.1	22.2	2.7
Fukuoka	633	928	512	0	0	2,073	1.4	2.0	0.2	0.0	0.0	0.4
Saga	9,065	4,561	45,282	2,460	0	61,368	88.5	68.8	86.4	8.8	0.0	62.9§
Nagasaki	3,916	3,220	1,951	44	124	9,255	26.8	29.3	2.4	0.1	19.3	6.0
Kumamoto	9,072	2,119	367	182	65	11,805	39.4	18.4	0.4	0.3	9.5	6.1
Oita	1,137	376	83	59	0	1,655	11.9	3.7	0.1	0.2	0.0	1.4
Miyazaki	5,074	2,079	1,342	314	43	8,852	38.6	28.2	2.0	0.9	6.7	7.2
Kagoshima	4,303	1,642	507	0	389	6,841	25.6	11.4	0.5	0.0	38.1	3.8
Okinawa	5,384	247	1,138	495	0	7,264	44.0	1.6	1.1	1.0	0.0	4.0
Total	123,604	65,182	517,247	68,095	3,468	777,596	11.3	5.4	7.3	1.9	5.6	6.0
Nationwide total	1,095,808	1,214,345	7,063,606	3,612,747	61,858	13,048,364						

§ *P* < 0.01.

**Figure 3:**
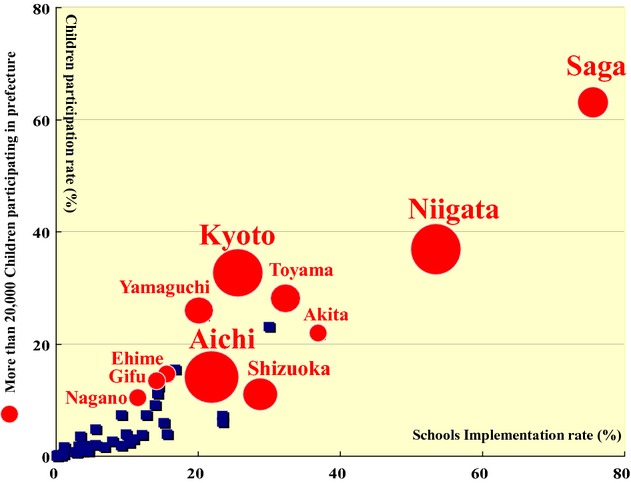
Schools implementation rate and children participation rate of school-based fluoride mouth-rinsing programme (S-FMR) in all 47 prefectures in Japan (2010).

Prefectures with an S-FMR implementation rate of at least 25% of all target schools in the area, in descending order, were Saga at 76%, Niigata at 54%, Akita at 37%, Toyama at 33%, Shimane at 30%, Shizuoka at 28% and Kyoto at 25%. In terms of participation rate, the highest was Saga at 63% of all target children, followed by Niigata at 37%, Kyoto at 33%, Toyama at 29% and Yamaguchi at 25% (*Figure* 3). The implementation and participation rates were particularly high in Saga Prefecture and were significantly higher than those of the other prefectures (*P* < 0.01; for both implementation rate and participation rate).

Analysis of inter-prefecture disparity from the cumulative relative frequency in number of schools implementing the S-FMR revealed that eight, or about 17%, of the 47 prefectures were in the top 50% for cumulative relative frequency in number of schools implementing S-FMR (261 or more schools), while 21 prefectures, or about 45%, were in the top 80% for cumulative relative frequency in number of schools implementing S-FMR (regions with 114 or more schools). Similar analysis on number of participants revealed that five, or about 10%, of prefectures were in the top 50% for cumulative relative frequency in number of children participating in the S-FMR (40,694 or more children), while 16 prefectures, or about 34%, were in the top 80% for cumulative relative frequency in number of children participating in S-FMR (regions with 11,881 or more children). This means that about 10–20% of prefectures account for roughly half of the schools implementing S-FMR and children participating in the programme, showing a clear disparity between prefectures.

Looking at the number of implementing schools by type, 10–20% of prefectures account for roughly half of all schools implementing the S-FMR in Japan: 1,756 preschools (48% of total) in Niigata, Aichi, Shizuoka, Kumamoto, Hyogo and Saga (regions with 205 or more preschools); 523 kindergartens (51%) in Shizuoka, Aichi, Saga, Hyogo, Miyagi, Niigata, Nagasaki and Chiba (regions with 41 or more kindergartens); 1,183 elementary schools (50%) in Niigata, Aichi, Kyoto, Saga and Shimane (134 or more elementary schools); and 194 junior high schools (50%) in Niigata, Akita, Shimane, Yamaguchi and Saga (24 or more junior high schools). Similarly, looking at the number of children participating in the programme by type of school, only about 10% of prefectures account for roughly half of all children participating S-FMR in Japan: 64,956 children in preschools (53% of total) in Niigata, Shizuoka, Aichi, Kumamoto, Saga and Hyogo (regions with 8,225 or more children); 34,545 children in kindergartens (53%) in Shizuoka, Aichi, Saga, Nagasaki, Miyagi, Chiba and Hokkaido (regions with 3,102 or more children); 278,165 children in elementary schools (54%) in Aichi, Kyoto, Niigata and Saga (45,282 or more children); and 35,088 children in junior high schools (52%) in Niigata, Nagano, Akita, Gifu and Yamaguchi (3,928 or more children).

In Japan, a total of 690 municipalities (39%) were implementing S-FMR. Looking at the results for each prefecture showed that the top prefectures – Saga, Kagawa, Ehime and Niigata – accounted for over 90% of municipalities implementing the programme. At least one school in almost all municipalities in these prefectures was implementing the programme. In contrast, <5% of municipalities were implementing the programme in Tokyo, Okayama and Osaka, which were the three prefectures with the lowest implementation rates.

*Table* **3** shows the number of schools implementing fluoride mouth-rinsing in each type of school and frequency per week, mouth-rinse fluoride concentration, type of agent and S-FMR funding party. The percentage of children rinsing five times a week and once a week was 66% and 20%, respectively, in preschool, and 61% and 23%, respectively, in kindergarten, with five times a week being more common in both types of school. In contrast, less than 3% and 96% of elementary school and junior high school, and 39% and 59% of schools for special needs education rinsed five times a week and once a week, respectively, with once a week being more common for all these types of school. The ratio of rinsing once a week rose with increasing age of children in schools. With regard to other frequencies for fluoride mouth-rinsing, less than 14% of preschool and kindergarten and 1% of elementary school rinsed twice a week.

**Table 3:** Outline of the rinsing methods and responsible financial support by type of school in the questionnaire for school-based fluoride mouth-rinsing programme (S-FMR) in Japan (2010)

Questionnaire	Choices	Preschool (%)	Kindergarten (%)	Elementary school (%)	Junior high school (%)	School for special needs education (%)	Total (%)
Frequency per week	Five times a week	66.4	60.5	2.4	2.8	39.0	40.0
	Two times a week	12.3	13.9	1.0	0.6	0.0	7.9
	Once a week	19.7	23.1	96.2	96.3	58.5	50.8
	Other	1.6	2.5	0.3	0.3	2.4	1.2
Fluoride Concentration	225–250 ppm F	67.4	60.7	3.0	4.5	25.6	40.9
	450 ppm F	23.8	24.6	23.6	18.7	9.3	23.5
	900 ppm F	6.4	9.9	71.0	72.9	58.1	32.8
	Other	2.5	4.8	2.3	3.9	7.0	2.9
Agent used in S-FMR	Sodium fluoride reagent	14.6	26.0	63.3	70.3	41.3	35.7
	Fluoride mouth-rinse ethical drug (MIRANOL and ORA-BLISS)	84.1	72.6	36.5	29.4	58.7	63.4
Responsible for financial support	Local public body and board of education	70.9	67.2	77.0	83.9	63.0	73.1
	School	11.9	14.5	2.2	2.9	21.7	8.6
	Parent	8.8	8.8	14.1	9.4	6.5	10.6
	Regional dental association	3.9	3.6	2.3	0.9	4.4	3.1
	Other	4.5	5.9	4.4	2.9	4.4	4.6

The most common mouth-rinse fluoride concentration was 225–250 ppm F for preschool and kindergarten (67% and 61%, respectively) and 900 ppm F for elementary school, junior high school and schools for special needs education (71%, 73% and 58%, respectively). The ratio of 900 ppm F concentration rose with increasing age, similar to the frequency of rinsing once a week. Other fluoride concentrations, such as 450 ppm F and 100 ppm F, were also used.

With regard to the type of fluoride mouth-rinsing agent used, sodium fluoride reagent and fluoride mouth-rinse ethical drug were used by 15% and 84% of preschool, respectively, and by 26% and 73% of kindergarten, 63% and 37% of elementary school, 70% and 29% of junior high school, and 41% and 59% of schools for special needs education. The ratio of rinsing with sodium fluoride reagent rose with increasing age of children of schools.

The funding party or organisation was most often the government and board of education, (71% for preschools, 67% for kindergartens, 77% for elementary schools, 84% for junior high schools and 63% for schools for special needs education). The results suggest that S-FMR at preschools, kindergartens and schools for special needs education are funded by the school itself or by the parents.

*Table* **4** shows the status of fluoride application measures in the health-promotion plans of local public bodies. All 47 prefectures and 89 cities with public health centres had formulated health-promotion plans. Although fluoride application for caries prevention was included in 77% of health promotion plans, 6% did not include any detailed means for such fluoride application. Only 33% of plans noted fluoride mouth-rinsing as a method for application, which was significantly lower than mentions of fluoride painting and fluoride dentifrice in 60% and 54% of plans, respectively (*P* < 0.01).

**Table 4:** Availability of fluoride application programme in health-promotion plans of local public bodies in Japan (2010)

Classification of local public body*	Available fluoride application programme in health promotion plan	Available fluoride gel in health-promotion plan	Available fluoride dentifrice in health-promotion plan	Available fluoride mouth-rinsing in health promotion plan
Prefecture (47)†	85.1% (40)	76.6% (36)	61.7% (29)	55.3% (26)
Special ward of Tokyo Metropolitan (23)	73.9% (17)	43.5% (10)	52.2% (12)	8.7% (2)
Ordinance-designated city (18)	88.9% (16)	83.3% (15)	66.7% (12)	33.3% (6)
Municipality; Core city (41)	68.3% (28)	43.9% (18)	48.8% (20)	24.4% (10)
Public health centre ordinance-designated city (7)	57.1% (4)	28.6% (2)	14.3% (1)	14.3% (1)
Total (136 local public bodies)*	77.2% (105)	59.6% (81)	53.7% (73)	33.1% (45)

* The number of local public bodies as of March 2010 in Japan.

† (): Number of local public bodies.

## Discussion

Fluoride mouth-rinsing is very effective for preventing dental caries, is both safe and easy to apply and is a public health method based on scientific evidence^11^–^18^. For these reasons, it is estimated that fluoride mouth-rinsing is being performed in groups and at home by about 100 million people throughout the world^19^,^20^. In particular, reports have claimed that fluoride mouth-rinsing for preschool children is very safe in Japan compared with Western countries^1^–^6^, and it is recommended that children begin fluoride mouth-rinsing either through the S-FMR or at home, from age 4 or 5 years^5^,^6^. A WHO website (WHO Bank of Ideas) also states that the S-FMR performed at preschools is extremely beneficial for countries such as Japan that do not fluoridate the water^21^. The website also contains information on the status of S-FMR adoption by groups of preschool children, implementation procedures (with pictures), volume of post-rinsing residue and preventive effects, and gives examples of implementing the programme under appropriate management^21^,^22^. Fluoride mouth-rinsing is thus an important means for preventing caries in permanent teeth in almost all life stages in Japan and the S-FMR should be more widely carried out as an important public measure for children in particular^1^–^6^,^23^.

A look at recent trends regarding fluoride application in Japan shows that various relevant organisations such as MHLW, the Japan Dental Association, the Japanese Association for Dental Science and the Japanese Society for Oral Health are offering opinions and declarations on the subject, and are coming to recommend fluoride mouth-rinsing^1^,^2^. After MHLW published Fluoride Mouth-rinsing Guidelines (January 2003), S-FMR was adopted in Tokyo Metropolis and Nara Prefecture in 2005 and finally by the remainder of the 47 prefectures in Japan^1^,^2^,^4^. The efficacy of the S-FMR in Japan has been demonstrated in Niigata Prefecture, where it was implemented as early as 1970^1^–^6^,^23^. In FY 2006, Niigata Prefecture reached its goal for mean decayed/missing/filled teeth (DMFT) for 12-year-olds (1.0 or fewer teeth), as outlined in the Healthy Japan 21 campaign and, as of FY 2012, Niigata has maintained the smallest 12-year-old DMFT score of all prefectures in Japan for 13 years running^23^.

At a budget committee meeting of the House of Representatives, the Head of the Health Policy Bureau of MHLW praised the strong caries prevention effects and excellent public health characteristics of fluoride mouth-rinsing, based on the Fluoride Mouth-rinsing Guidelines^24^. Regarding the safety of fluoride mouth-rinsing, he expressed the opinion that safety is assured, as the rinse poses no health hazard even if swallowed completely, and regarded the possibility of collaboration with MEXT, saying that the Ministry intends to pursue dental health measures in the future through information exchange and other types of cooperation with MEXT^24^. As a result, the Act Concerning the Promotion of Dental and Oral Health was established as a national law in August 2011^25^. Building on the Health Promotion Act established in 2002, prefectural and municipal health promotion plans are being pursued based on scientific evidence. Concrete indicator (1) within the goals for preventing dental diseases in school-aged children, which is a basic matter in this Act, specifies that there should be an increase in the ratio of 12-year-olds without dental caries to a target of 65% (by FY 2022). The reasoning for this view is that ‘with changes in background factors such as fluoride dentifrice achieving a roughly 90% market share and user ratio, it is possible that the upward trend in ratio of children without caries may be limited. Moreover, it is important to take into account the current state of health-care activities related to promoting dental and oral health in schools. One of the targets related to dental and oral health in the second Healthy Japan 21 that began in April 2013 is to increase the number of infants and school-aged children without caries. In the section on future measures that are required, it states that there is a need to reduce health inequalities, including regional differences, and local public bodies (omission) need to implement caries prevention methods that have well-established evidence, such as fluoride application and pit-and-fissure sealant, as the local situation demands'. The process in Japan to date and case examples may serve as references for local public bodies to formulate measures and may serve an important role in reducing regional differences in the S-FMR. Future measures will further advance the S-FMR that has guaranteed safety and beneficial effects.

Based on the results of the present study, implementation of S-FMR is still low in all types of schools^4^. Implementation rates and participation rates are even lower in elementary and junior high schools when compared with preschools and kindergartens. One factor accounting for the higher implementation rate in preschools and kindergartens may be that it is being pursued on the basis of safety^1^–^6^. While it is easy for preschools and kindergartens to implement the S-FMR, increasing implementation rates at elementary and higher level schools remains a challenge. In regions where there are obstacles hindering adoption of the S-FMR in schools, efforts to consider the need for organizational involvement such as cooperation with government-related and other parties concerned with school dental health and the importance of educating dental professionals and local residents, spreading the adoption of S-FMR and reducing inter-prefecture differences can be linked to promoting lifelong oral health^1^,^2^,^4^. It may be necessary to approach local public bodies and school boards to encourage cooperation between MHLW and MEXT and effectively implement S-FMR during the life stages at which the risk of caries is greatest^24^.

There were also signs of inter-prefecture differences, as 10–20% of local public bodies accounted for the top 50% of S-FMR implementation rates and 35–45% of local public bodies accounted for the top 80% of S-FMR participation rates. This shows both inter-prefectural differences and the implementation rates of different municipalities in a single region.

One reason for these differences may be the presence or absence of fluoride application measures in health-promotion plans of the local public bodies. In particular, mention of fluoride mouth-rinsing was not common, at only 33% overall, which was significantly lower than mentions of fluoride painting (60%) and of fluoride dentifrice (54%). This result stems largely from the low rate in ordinance-designated cities with public health centres when compared with prefectures.

In order to adopt caries prevention measures as public health policy, local public bodies must plan sustainable methods as a priority and expand measures that are highly cost-effective. Reducing differences in the extent of fluoride mouth-rinsing adoption among prefectures that consider S-FMR a measure to focus on, and having the government act as the main party to provide organisational, financial and environmental support may largely contribute to the advance of fluoride mouth-rinsing^1^,^2^,^4^.

As a measure that must be enforced by the government, S-FMR is recognised as a programme with good organisational sustainability and consistency from example cases in Japan. As it is clear that there is a public responsibility to implement the S-FMR, there is good potential for receiving public works funding. Achieving lifelong oral health in addition to caries prevention requires consideration of various criteria for the benefit of regions, such as the positioning of oral health policies, cooperation within government sectors, cooperation with regional dental associations, consensus building among regional parties concerned and social support that includes a combination of elements such as provision of information to local residents and educational support^4^. It may be effective for local public bodies in Japan to clearly state the S-FMR targets shown in this survey in their health promotion plans.

Finally, social support is essential for achieving good lifelong oral health. Local public bodies and their boards of education, as well as dental associations and other regional health-related organisations, can play an important role in implementing and sustaining S-FMR, and in reducing health inequalities concerning caries in permanent teeth (among regions and individuals). In Japan, an urgent response is needed to eliminate the increasing gap between prefectures.
